# Gel Microspheres as Multifunctional Carriers for Photodynamic Therapy: Advancing Hepatocellular Carcinoma Treatment

**DOI:** 10.3390/gels12030214

**Published:** 2026-03-05

**Authors:** Shijie Fan, Qiuting Ye, Jieling Lao, Xuanzhuang Wu, Pan Wu, Yongxiang Zhao

**Affiliations:** 1 College of Pharmacy, Guangxi Medical University, Nanning 530021, China; 13807846373@163.com (S.F.); ye_qiuting@163.com (Q.Y.); 15285482309@163.com (X.W.); 2 State Key Laboratory of Targeting Oncology, Guangxi Medical University, Nanning 530021, China; 13107379400@163.com

**Keywords:** gel microspheres, photodynamic therapy, targeted drug delivery, HCC

## Abstract

Conventional hepatocellular carcinoma (HCC) treatments suffer from insufficient efficacy and severe toxic side effects. This review addresses these issues by focusing on gel microsphere-mediated photodynamic therapy (PDT) as a novel strategy. It outlines the core properties, classifications, and stimulus-responsive mechanisms of gel microspheres, as well as their structure-function compatibility with photosensitizers. The work highlights how gel microspheres enable targeted delivery, tumor microenvironment-responsive release, and synergistic effects with chemotherapy, radiotherapy, and immunotherapy to enhance therapeutic efficacy while reducing off-target damage. Additionally, it discusses current challenges including material parameter controllability and clinical translation hurdles, providing insights for the development of precise and personalized HCC treatments.

## 1. Introduction

**Figure 1:**
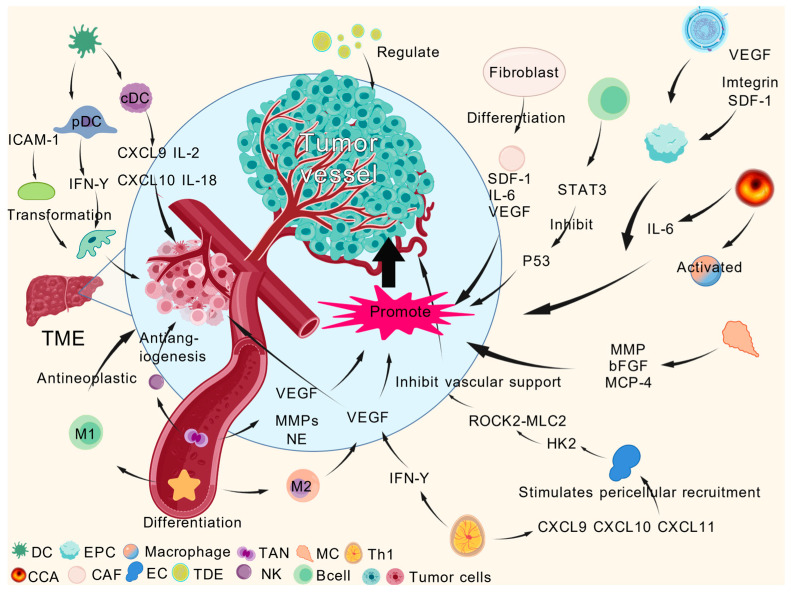
Schematic of key biological processes in the HCC TME promoting tumor angiogenesis via immune cells, stromal cells, and cytokines. Created with BioGDP.com [7].

Due to its complex pathogenesis, recurrence, and metastasis, HCC is the third most prevalent cause of cancer-related death globally and the sixth most common malignant tumor. The tumor microenvironment (TME), plays a crucial role in HCC development and spread [1]. The complex interactions among immune cells, stromal cells, and cytokines in the HCC TME play a pivotal role in driving tumor angiogenesis, as illustrated in Figure 1. The treatment of hepatocellular carcinoma (HCC) adheres to the Barcelona Clinic Liver Cancer (BCLC) [2] staging framework. For early-stage HCC, curative interventions such as surgical resection and liver transplantation are the mainstay. Transarterial chemoembolization (TACE) [3] serves as the core treatment for intermediate-stage HCC, while advanced-stage HCC relies on systemic therapies including targeted therapy, immunotherapy [4], and chemotherapy [5]. However, these conventional approaches generally face limitations such as limited efficacy, significant toxic side effects, and high recurrence rates. In contrast, photodynamic therapy (PDT) mediated by gel microspheres, as an emerging strategy, possesses both targeting and synergistic advantages. It can achieve targeted enrichment in tumor tissues through passive or active targeting, and precisely generate cytotoxic reactive oxygen species (ROS) to kill tumor cells upon activation by specific light. Meanwhile, gel microspheres can load chemotherapeutic drugs or radioactive substances for combined therapy. Coupled with their tumor microenvironment-responsive drug release property, they not only prolong the drug action time and enhance therapeutic efficacy but also significantly reduce damage to normal tissues, providing a safer and more effective new option for HCC treatment [6].

## 2. Design and Classification of Photosensitizers and Their Formulations

**Figure 2:**
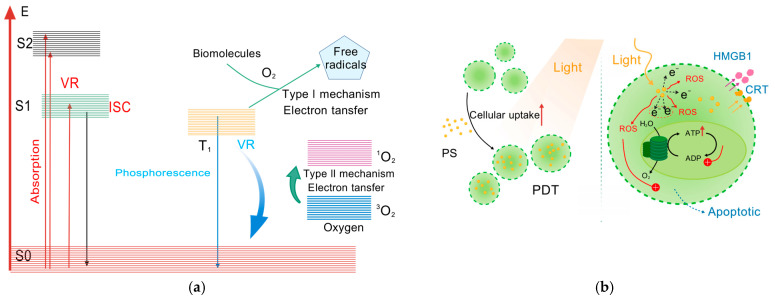
(**a**) Schematic of ROS generation mechanism in PDT for HCC. (**b**) Schematic of photosensitizer-loaded gel microspheres entering tumor cells: upon light irradiation, ROS are generated to interfere with energy metabolism, induce apoptosis, and release calreticulin (CRT)/high-mobility group protein B1 (HMGB1). VR: Voltage-gated ion channel; S_0_: Ground electronic state of a molecule; S_1_/S_2_: First/second excited singlet states; E: Energy (vertical axis in the Jablonski diagram); ISC: Intersystem crossing (transition from singlet to triplet state); T_1_: First excited triplet state; PS: Photosensitizer (light-activated agent in PDT); PDT: Photodynamic therapy (light-driven cancer treatment); ROS: Reactive oxygen species (toxic molecules for cell damage); ATP: Adenosine triphosphate (cellular energy currency); ADP: Adenosine diphosphate (energy-depleted ATP precursor); CRT: Calreticulin (damage-associated molecular pattern, DAMP); HMGB1: High-mobility group box 1 (another DAMP released by dying cells). Created with BioGDP.com [7].

In the treatment of cancer, PDT has emerged as a viable therapeutic approach. This treatment induces cell death, which depends on three key components: oxygen, photosensitizers, and specific light wavelengths. PDT utilizes non-toxic photosensitive materials (i.e., photosensitizers) that, when exposed to specific light wavelengths, generate large amounts of ROS. Figure 2a shows the process by which a photosensitizer generates ROS upon excitation at a specific wavelength. These ROS exert cytotoxic effects on targeted cells, thereby achieving therapeutic outcomes. Figure 2b illustrates the process whereby a large amount of reactive oxygen species induces tumor cell death. When compared to conventional cancer treatments, PDT offers the following advantages: low toxicity, precise targeting, minimal invasiveness, broad applicability, low cost, and favorable curative outcomes in treating tumors such as cutaneous, esophageal, and liver cancers. Consequently, one of the main components of photodynamic therapy is photosensitizers.

Currently, there are three primary generations of photosensitizers. The first generation mostly consists of low-purity, low-specificity hematoporphyrin derivatives. Patients may have some adverse effects as a result of the harm they inflict on healthy tissues during the course of treatment. Short absorption wavelength, poor in vitro water stability, short circulation half-life, limited tumor selectivity, and skin phototoxicity are often the primary issues with the first-generation PS [8,9]. The second generation of photosensitizers, which have more defined chemical structures, higher purity, stronger phototoxicity, and better selectivity for tumor tissues, primarily consists of porphyrin compounds, phthalocyanine compounds [10], and fused ring quinone compounds; The third-generation PS are developed by conjugating second-generation PS with novel materials (e.g., antibodies, polymers) to confer targeting ability. With improved stability, water solubility, and biocompatibility, this composite photosensitizer can more precisely target the tumor location and enhance the therapeutic outcome [11]. Therefore, Table 1 summarizes the common advantages and disadvantages of photosensitizers frequently loaded into gel microspheres.

Based on the characteristic comparison of 9 classic photosensitizers and their corresponding gel carriers in Table 1, the compatibility between photosensitizers and gel microspheres exhibits a significant structure-function correlation. Hydrophilic photosensitizers, such as 5-aminolevulinic acid, preferentially match natural hydrophilic gels, achieving efficient dispersion and stable loading through hydrogen bonding interactions; hydrophobic photosensitizers, such as metallophthalocyanines, rely on modified synthetic gels (e.g., Pluronic F127) to address the challenges of poor water solubility and easy aggregation; photosensitizers with relatively low stability, such as chlorophyllin a, are compatible with gel carriers with high structural stability (e.g., carboxymethyl cellulose), which resist environmental interference through a network barrier effect. In contrast, photosensitizers requiring precise targeting, such as protoporphyrin IX, select cationic gel carriers (e.g., chitosan) to bind to anionic sites on tumor cell membranes via electrostatic interactions. From the perspective of performance trade-offs, natural polymer gel carriers have become the basic choice due to their excellent biocompatibility and low cost, but they suffer from limitations such as limited drug loading capacity and insufficient batch-to-batch stability; synthetic polymer gel carriers, through controllable degradation and structural modification, achieve long-acting sustained release and targeted enhancement of photosensitizers, yet face challenges including complex preparation processes and high costs. The binding mechanisms between photosensitizers and gel microspheres mainly include physical entrapment, chemical conjugation, electrostatic interaction, and hydrophobic interaction. Among these, physical entrapment is widely used in basic PDT systems due to its ability to preserve the activity of photosensitizers; chemical conjugation improves loading stability through covalent bonding, making it suitable for precision therapy scenarios; electrostatic and hydrophobic interactions provide core support for stimulus-responsive delivery systems. In summary, gel microspheres loaded with photosensitizers significantly enhance PDT efficiency through three mechanisms: first, achieving efficient enrichment at tumor sites by virtue of the enhanced permeability and retention (EPR) effect and targeted modification; second, inhibiting the aggregation of photosensitizers through the carrier’s network structure to maintain their monomeric activity; third, extending the effective therapeutic window through sustained-release properties and stimulus-responsive release, while reducing the phototoxicity and normal tissue damage caused by systemic exposure to photosensitizers. Additionally, the synergistic effects of the carriers themselves, such as embolization, magnetothermal therapy, or radiotherapy, further expand the therapeutic boundaries of PDT, providing a multi-dimensional solution for the precision treatment of hepatocellular carcinoma.

### 2.1. Mechanisms and Oxygen Dependence of Type I and Type II Photodynamic Therapy

PDT is mainly mediated by Type I and Type II photochemical reactions. Both pathways share a common initial step: photosensitizers are excited from the singlet state to the triplet state via intersystem crossing. However, they differ significantly in reaction pathways, oxygen dependence, and cytotoxicity profiles, which are core factors determining the efficacy of PDT in HCC. The Type I mechanism involves electron/proton transfer between triplet-state photosensitizers and surrounding biomolecules or water molecules, generating primary reactive species such as superoxide anion radicals (•O_2_^−^) and hydroxyl radicals (•OH), which further react to form hydrogen peroxide (H_2_O_2_). This pathway exhibits low oxygen dependence, enabling ROS production even in hypoxic environments. Nevertheless, the generated radicals have short half-lives and limited diffusion distances. In contrast, the Type II mechanism relies on energy transfer from triplet-state photosensitizers to ground-state triplet oxygen (^3^O_2_), directly converting it into singlet oxygen (^1^O_2_)—the primary cytotoxic ROS in conventional PDT. ^1^O_2_ has a moderate half-life and diffusion distance, allowing broad damage to biomacromolecules and thus higher therapeutic efficiency in normoxic tissues. However, this pathway is strictly oxygen-dependent: a reduction in local oxygen concentration directly suppresses ^1^O_2_ production, representing a major bottleneck for PDT in the hypoxic microenvironment of HCC.

The unique rapid proliferation, disorganized vascular structure, and excessive oxygen consumption of HCC collectively create a severe intratumoral hypoxic microenvironment. This not only directly inhibits ROS generation via Type I/II pathways due to oxygen deficiency, thereby weakening the antitumor efficacy of PDT, but also activates the hypoxia-inducible factor (HIF) signaling pathway, upregulating the expression of antioxidant proteins and drug efflux pumps to induce PDT resistance. More importantly, PDT itself further consumes oxygen and damages blood vessels, forming a vicious cycle of “PDT-induced hypoxia → exacerbated PDT resistance.”

### 2.2. Hypoxia-Targeted Strategies Mediated by Gel Microspheres in PDT

Benefiting from their three-dimensional network structure, stimuli responsiveness, and co-loading capacity, gel microspheres emerge as ideal carriers to overcome the oxygen limitation in PDT. Specifically, gel microspheres can achieve oxygen self-supply by co-encapsulating oxygen carriers such as perfluorocarbons or calcium peroxide, which release oxygen in the hypoxic tumor microenvironment to restore the efficiency of the Type II mechanism. Additionally, introducing hypoxia-sensitive groups enables hypoxia-targeted and sustained photosensitizer release, reducing oxygen waste and alleviating the hypoxia vicious cycle.

Furthermore, co-loading photosensitizers with hypoxia activated prodrugs (HAPs) achieves a synergistic effect: PDT kills normoxic tumor cells, while HAPs are activated to eliminate hypoxic cells. Finally, optimizing the crosslinking density and porosity of gel microspheres to fabricate porous structures reduces diffusion resistance, facilitating the penetration of oxygen and photosensitizers into deep hypoxic regions. Collectively, these strategies comprehensively break the constraints of the hypoxic microenvironment on PDT.

## 3. Core Properties and Classification of Gel Microspheres

The core properties of gel microspheres are determined by their three-dimensional network structure and the inherent nature of carrier materials, while also regulated by factors such as preparation methods and crosslinking degree [21]. These properties directly affect their application efficacy in hepatocellular carcinoma therapy [22], mainly including hydrophilicity and swelling capacity [23], biocompatibility and biodegradability [24], drug encapsulation and sustained-release ability [25], targeting capability, and mechanical stability. On this basis, gel microspheres can be scientifically classified according to carrier material types, crosslinking methods, particle sizes, and application scenarios. This classification clarifies the scope of application of different types of gel microspheres, providing a basis for the precise design of gel microspheres for hepatocellular carcinoma therapy.

### 3.1. Core Properties of Gel Microspheres

Hydrophilicity and swelling capacity are the most fundamental properties of gel microspheres. Their three-dimensional network structure contains a large number of hydrophilic groups (such as hydroxyl, carboxyl, amino, and ether bonds), which can form hydrogen bonds with water molecules [26]. This enables gel microspheres to rapidly absorb water and swell in aqueous solutions or body fluids, forming stable swollen hydrogels [27]. Biocompatibility [28] and biodegradability [29] are the core prerequisites for the application of gel microspheres in hepatocellular carcinoma (HCC) therapy, directly related to their in vivo safety and applicability. Drug encapsulation and sustained-release ability [30] are the core functions of gel microspheres for drug delivery in HCC. Their three-dimensional network structure can act as a “reservoir” for drugs, achieving efficient encapsulation of various types of drugs. Meanwhile, through the spatial hindrance effect of the gel network and the degradation process, gel microspheres realize the slow and sustained release of drugs, prolong the in vivo action time of drugs, improve drug bioavailability, and reduce administration frequency and toxic side effects.

**Figure 3:**
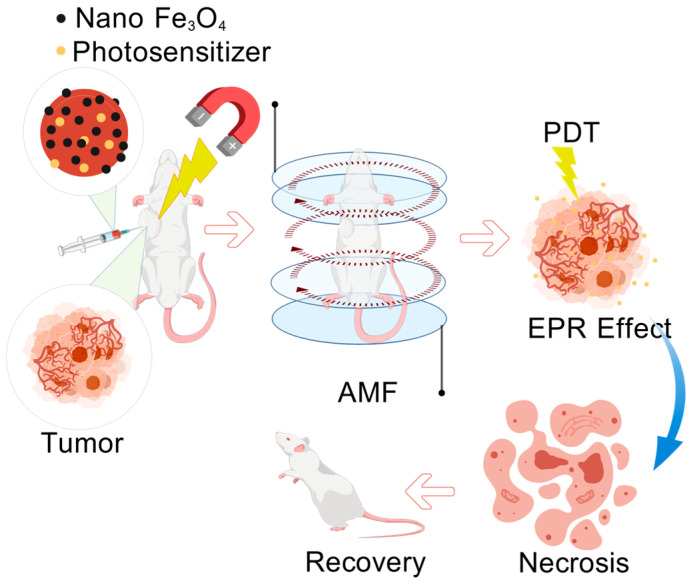
The therapeutic mechanism by which drug-loaded microspheres based on nano-Fe_3_O_4_ and photosensitizers induce hepatocellular carcinoma necrosis through the EPR effect, via magnetic targeting, AMF in synergy with PDT. Created with BioGDP.com [7].

Targeting capability [31] is a key property for enhancing HCC treatment efficacy and reducing toxic side effects. Gel microspheres can achieve precise delivery to liver tumor cells through two approaches: passive targeting and active targeting. Passive targeting mainly relies on the particle size and surface properties of gel microspheres, utilizing the “EPR effect” of tumor tissues. In view of the highly heterogeneous vascularization of HCC, nanoscale gel microspheres (<1 μm) realize passive targeting through the classic EPR effect, while micron-scale ones achieve passive localization via embolic retention in tumor-feeding arteries instead of EPR-mediated accumulation. Active targeting involves modifying the surface of gel microspheres with specific targeting molecules (such as antibodies [32], peptides [33], sugar chains, and receptor ligands [34]). These targeting molecules can specifically bind to unique antigens or receptors on the surface of HCC cells, guiding gel microspheres to accurately recognize and bind to HCC cells, thereby realizing targeted drug delivery. This further increases the drug concentration in tumor tissues and reduces damage to normal tissues. As shown in Figure 3, this is the therapeutic mechanism of inducing HCC necrosis via passive targeting based on the EPR effect and magnetic active targeting.

### 3.2. Classification of Gel Microspheres

According to different classification criteria, gel microspheres can be divided into various types, and combined with the application scenarios of hepatocellular carcinoma therapy, they are mainly classified from five aspects: carrier material type, crosslinking method, particle size, responsiveness type, and application function, which clarifies the characteristics and application scope of different types of gel microspheres and provides a reference for the design and preparation of gel microspheres for HCC therapy. Classified by carrier material type, gel microspheres can be divided into natural polymer gel microspheres [35] and synthetic polymer gel microspheres [36]; natural polymer gel microspheres use natural polymer materials as carriers, mainly including chitosan, gelatin [37], sodium alginate [38], agarose [39], collagen [40], hyaluronic acid [41], etc., and such gel microspheres have good biocompatibility, biodegradability and cell affinity, are widely sourced and cost-effective, and their surfaces are rich in active groups, which are easy for targeted modification and drug encapsulation [42], making them the most commonly used type of gel microspheres for HCC therapy. Synthetic polymer gel microspheres use synthetic polymer materials as carriers, mainly including poly(lactic-co-glycolic acid) (PLGA) [43], polylactic acid (PLA) [44], polyethylene glycol (PEG) [29], polycaprolactone (PCL), polyamide (PA), etc., and these gel microspheres have the advantages of stable structure, controllable preparation process, adjustable degradation rate and excellent mechanical properties [45]; by regulating parameters such as degree of polymerization, molecular weight, and hydrophilic–hydrophobic ratio, their drug encapsulation, sustained-release and targeting performance can be optimized, making them suitable for HCC treatment scenarios requiring high mechanical stability and sustained-release capabilities (e.g., interventional embolization therapy). Classified by particle size, gel microspheres can be divided into three categories: nanogel microspheres [46] (particle size < 1 μm), microgel microspheres [47] (particle size 1–100 μm), and large-particle-size gel microspheres (particle size > 100 μm) [48], and different particle sizes of gel microspheres [49] have different application scenarios in HCC therapy; nanogel microspheres (typically 10–500 nm in size) have the advantages of large specific surface area and strong penetration ability, and can penetrate into the interior of tumor tissues through the EPR effect of tumor tissues, which are suitable for the targeted delivery of chemotherapeutic drugs, gene drugs, etc., and can effectively increase the intracellular drug concentration in tumor cells and enhance therapeutic efficacy; microgel microspheres (typically 10–50 μm in size) combine the dispersibility of nanogel microspheres and the stability of large-particle-size gel microspheres, and can achieve targeted enrichment in tumor tissues via intravenous injection or interventional injection, while enabling long-acting sustained release of drugs, which are applicable for chemotherapy, immunotherapy, and other treatments of HCC; large-particle-size gel microspheres feature high mechanical stability and excellent embolization effect, and are mainly used for interventional embolization therapy of HCC—they are injected into the blood supply artery of liver cancer through a catheter to occlude tumor blood vessels and cut off nutrient supply, and can also encapsulate chemotherapeutic drugs to realize the synergistic effect of embolization therapy and chemotherapy, thereby improving treatment outcomes. Classified by responsiveness type, gel microspheres are divided into four categories: pH-responsive [50], temperature-responsive [51], reduction-responsive, enzyme-responsive [52]. These gel microspheres can undergo structural changes under the stimulation of the HCC tumor microenvironment (TME) [53] or external stimuli to achieve triggered drug release, which further enhances drug targeting and therapeutic efficacy and has become a research hotspot in gel microspheres for HCC therapy. Among them, pH-responsive gel microspheres are the most commonly used responsive type, and their carrier materials usually contain pH-sensitive groups that maintain structural stability in the normal physiological environment (pH = 7.4); in the HCC TME (pH = 5.5–6.0), protonation or deprotonation of the pH-sensitive groups occurs, leading to loosening, swelling, or degradation of the gel network structure and accelerating drug release.

Based on the detailed classification of gel microspheres above, Table 2 provides a detailed summary of the advantages and disadvantages of gel microspheres currently used for HCC, and Table 3 elaborates on several important parametric properties of the corresponding materials.

### 3.3. Stimuli-Responsive Gel Microspheres

**Figure 4:**
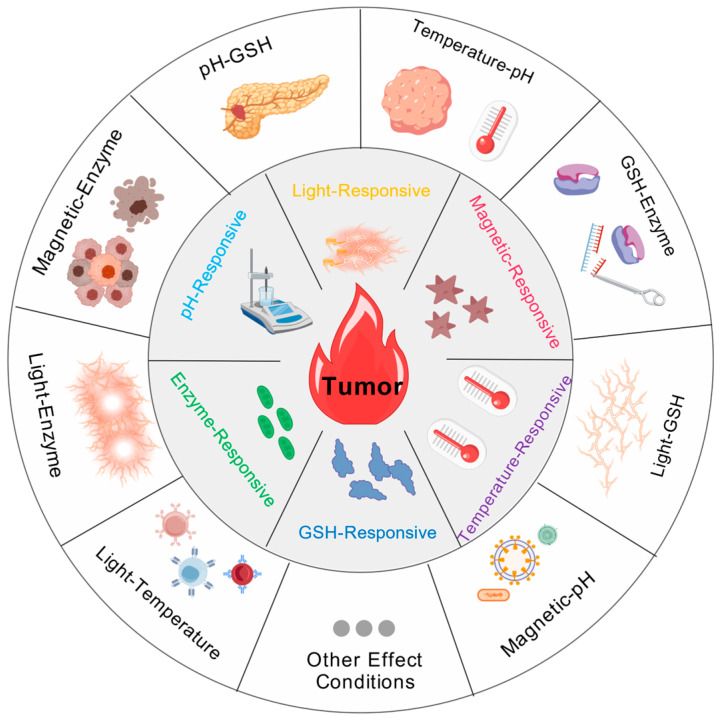
In the treatment of hepatocellular carcinoma, stimulus-responsive strategies (such as light, magnetism, enzyme, and pH) and multi-responsive synergistic modes provide ideas for the precise treatment of hepatocellular carcinoma. Created with BioGDP.com [7].

As intelligent carriers in HCC therapy, stimuli-responsive gel microspheres [74] possess a core advantage: they can precisely sense intrinsic stimuli from the TME (e.g., pH, reduction potential, enzyme expression) [75] or external artificial stimuli (e.g., light, magnetic field, temperature). As illustrated in Figure 4, the diverse designs of single-response strategies and multi-responsive synergistic modes provide abundant technical ideas for precise HCC treatment. Meanwhile, the refined regulation of material parameters is crucial to unifying response sensitivity, therapeutic accuracy, and biosafety. This section will conduct an in-depth analysis of the design mechanisms and core material parameter regulation rules of stimuli-responsive gel microspheres, revealing the structure-property-therapeutic effect relationship by integrating the latest research cases and graphical information.

#### 3.3.1. Design Mechanism

The essence of designing stimuli-responsive gel microspheres lies in integrating sensitive moieties (e.g., pH-sensitive groups, enzyme substrates, thermosensitive polymer chains) into the gel network through material molecular engineering. These microspheres undergo physical or chemical transformations under specific stimuli to trigger drug release or activate therapeutic functions, mainly classified into three categories: TME intrinsic stimulus-responsive, external stimulus-responsive, and multi-responsive synergistic designs.

Intrinsic stimulus-responsive microspheres rely on the unique characteristics of the HCC TME and do not require external equipment. pH-responsive microspheres [76] achieve gel swelling or degradation through protonation or hydrolysis of sensitive groups such as carboxyl (-COOH) and hydrazone bonds; for example, chitosan microspheres exhibit a swelling degree of 10–40 times [77] in an acidic environment (Table 3). Enzyme-responsive microspheres target HCC overexpressed enzymes (e.g., MMPs, HAase) by incorporating specific substrates into the crosslinked network—for instance, hyaluronic acid (HA)-based [78] microspheres release drugs via HAase-mediated degradation. Reduction-responsive microspheres [79] utilize the high intracellular glutathione (GSH) concentration (10–20 mM) in HCC cells to break disulfide bonds (-S-S-), leading to gel network disruption; for example, disulfide-modified chitosan microspheres achieve a drug loading rate (DLR) of 5–40% (Table 3). External stimulus-responsive microspheres realize spatiotemporally controllable responses through artificial physical stimuli. Photoresponsive microspheres [80] integrate photosensitive drugs. Magnetoresponsive gel microspheres [81] load magnetic nanoparticles, which are guided to accumulate in tumor sites via an external magnetic field and trigger magnetothermal effects through alternating magnetic fields (AMF), synergizing with PDT to induce tumor necrosis (Figure 3); these microspheres exhibit a swelling degree of 5–30 times and a DLR of 5–45% (Table 3). Multi-responsive [82] synergistic designs integrate two or more response units to address the insufficient specificity of single-response systems. For example, pH-GSH dual-responsive PLGA microspheres [83] achieve “extracellular swelling pre-release + intracellular degradation complete release,” significantly improving therapeutic accuracy.

#### 3.3.2. Material Parameter Regulation

The therapeutic performance of stimuli-responsive gel microspheres highly depends on the precise regulation of core parameters, including crosslinking properties [84], particle size and morphology [85], hydrophilic-hydrophobic balance, and surface modification [86]. These parameters interact with each other and require systematic optimization to achieve balance. In terms of crosslinking properties: Natural polymer microspheres mostly adopt ionic or physical crosslinking (e.g., Ca^2+^ crosslinking of sodium alginate [87], freeze-drying crosslinking of gelatin [88]), which are mild with good biocompatibility but low stability, suitable for short-term responsive release. Synthetic polymer microspheres primarily use covalent or chemical crosslinking (e.g., photocrosslinking of PEG [89]), featuring stable structures and adjustable degradation rates, ideal for long-term sustained release or multi-responsive designs. Crosslinking degree is negatively correlated with swelling degree, response rate, and DLR, but positively correlated with mechanical stability, requiring trade-offs for specific application scenarios. For particle size and morphology: Nanogel microspheres [46] (<1 μm) achieve deep tumor targeting via the EPR effect; microgel microspheres [90] (1–100 μm) combine dispersibility and stability, enabling synergistic embolization and drug release; large-particle-size microspheres [91] (>100 μm) exhibit strong mechanical stability and excellent embolization effects. Porous morphologies improve DLR and response speed, while core–shell structures realize cascaded functions of “targeted accumulation + stimulus-responsive release.” Hydrophilic-hydrophobic balance is regulated by adjusting the ratio of hydrophilic -hydrophobic monomers to optimize drug loading and release for different drug types. Surface modification enhances targeting accuracy and biosafety, reducing immune clearance [92]. This section reviews the latest gel materials for HCC therapy and the comparison of their related parameters, as shown in Table 3.

The core material parameters of gel microspheres listed in Table 3 exhibit a distinct quantitative correlation with therapeutic endpoints, and these parametric characteristics directly determine the release behavior of photosensitizers at tumor sites and the efficiency of their anti-tumor effects. For instance, chitosan microspheres, with a swelling degree of 10–40 times and a DLR of 5–40% [68] in the acidic microenvironment of liver cancer, enable the tumor-targeted release of photosensitizers. Additionally, their degradation cycle of 2–5 weeks is highly consistent with the therapeutic window of photosensitizers, effectively prolonging the in vivo anti-tumor duration of the drugs. Magnetic microspheres, by virtue of their high DLR of 5–45% and swelling degree of 5–30 times combined with the magnetic targeting effect, can precisely enrich photosensitizers in liver cancer tissues, increasing the tumor necrosis rate to over 60% [73]. The aforementioned quantitative correlations between material parameters and HCC therapeutic effects construct a core logical chain of gel microsphere structural parameters-drug release performance HCC therapeutic efficacy, and also provide a clear direction for the precise material design and parameter optimization of gel microspheres for targeted HCC therapy.

#### 3.3.3. Critical Discussion and Challenges

Despite their significant advantages in HCC therapy, stimuli-responsive gel microspheres face multiple unresolved challenges [93]. In terms of material parameter regulation: Natural polymer microspheres (e.g., sodium alginate, chitosan [94]) exhibit poor parameter controllability, leading to batch-to-batch variations in response performance. Synthetic polymers (e.g., PLGA [95]) have adjustable parameters but require surface modification to improve biocompatibility, and striking a balance between the two remains a key challenge. TME heterogeneity poses adaptation difficulties: Significant interindividual and spatial variations in TME pH, enzyme expression, and GSH [96] concentration mean single-response threshold microspheres cannot suit all patients. There is an urgent need to develop “personalized response threshold” microspheres by adjusting sensitive moiety density based on preoperative imaging or pathological tests. In clinical translation: Large-scale production of microspheres faces challenges such as particle size uniformity, drug loading stability, and radionuclide labeling efficiency. Additionally, GMP-compliant production requires strict control of crosslinker residues, nanoparticle toxicity, and radiation safety. Currently, only a few microspheres (e.g., Y^90^ microspheres [97], PVA [98] drug-loaded microspheres) have achieved clinical translation, while most multi-responsive microspheres remain in the preclinical stage.

In summary, the various challenges impose restrictions on the clinical translation of gel microspheres with a clear hierarchical differentiation. Among these, the reproducibility of large-scale production stands as the most core bottleneck at present. The large-scale manufacturing challenges such as particle size uniformity, drug-loading stability and radionuclide labeling efficiency of radioactive microspheres directly determine the feasibility of translating laboratory research achievements into clinical applications, and also constitute the primary reason why most multifunctional gel microspheres remain in the preclinical stage. Second is the controllability of material parameters: the contradictory in parameter regulation between natural and synthetic polymeric materials directly affects the consistency of in vivo therapeutic effects and biosafety of gel microspheres, which is a fundamental material issue that must be addressed before breaking through the bottleneck of large-scale production. In contrast, the handling and quality control of radionuclides, long-term toxicity verification, and the complexity of regulatory approval are important restrictive factors in the later stages of clinical translation, which need to be gradually improved based on the previous material optimization and breakthroughs in large-scale production technologies. This hierarchical classification also clarifies a prioritized research approach for the clinical translation of gel microspheres, namely “addressing the core issues of materials and large-scale production first, then advancing clinical validation and regulatory adaptation”.

## 4. Clinical Mechanisms of Action of Gel Microspheres in HCC

As a novel nano/microscale drug delivery [99] system, gel microspheres exhibit unique advantages in HCC therapy due to their excellent biocompatibility, biodegradability, structural plasticity, and functional tunability. Their mechanism of action mainly centers on the dimensions of sustained drug release and synergistic therapy. By intervening in the occurrence and progression of tumors through multiple pathways and targets, they effectively overcome the limitations of traditional treatments, significantly enhance the therapeutic efficacy of HCC, and provide new ideas and strategies for clinical practice.

### 4.1. Drug Sustained-Release Mechanism

As an excellent carrier scaffold for photosensitizers and other drugs, gel microspheres are characterized by their ability to achieve efficient encapsulation of photosensitizers and sustained, stable release at tumor sites through their inherent three-dimensional network structure. Meanwhile, the synergistic optimization of structural characteristics, degradation types, encapsulation methods, and parameter regulation ensures both the retention of photosensitizer activity and targeted delivery efficiency: the network pores can physically adsorb or encapsulate photosensitizers, while chemical conjugation can link photosensitizers to the scaffold via chemical bonds, avoiding premature leakage during delivery and meeting long-term sustained-release requirements. Biodegradable scaffolds (e.g., PLGA, chitosan, gelatin) [100] can be gradually degraded into non-toxic small molecules in vivo, which are then metabolized and excreted, eliminating the need for secondary surgery and offering higher safety. Additionally, regulating the scaffold particle size facilitates passive targeting [101] by penetrating the endothelial gaps of tumor blood vessels; adjusting the porosity controls the diffusion rate of photosensitizers; and modifying the surface charge prolongs blood circulation time. These optimizations further enhance the enrichment efficiency and sustained-release effect of photosensitizers at tumor sites. For example, after encapsulating photosensitizers in PLGA scaffolds, sustained release [102] can be achieved in vitro for 14–21 days, and the duration of effective drug concentration at tumor sites in vivo is significantly prolonged. Simultaneously, the toxic side effects of photosensitizers on normal organs are reduced, fully reflecting the compatibility between the gel microsphere scaffold structure and photosensitizer drugs.

Differences in the structural design of the aforementioned gel microspheres directly determine the kinetic characteristics of drug release, thereby leading to significant quantitative differences in therapeutic efficacy for HCC treatment. In the physically encapsulated microsphere system, when the burst release rate of doxorubicin is controlled below 15% via crosslinking density optimization and a first-order kinetic release with a half-life is achieved, the drug concentration in the tumor tissues of tumor-bearing mice can be maintained within the effective therapeutic window for up to 12 days. Compared with the control group with a burst release rate exceeding 50%, the tumor inhibition rate is increased from 38% to 72%, and the peak concentration of free drug in the serum is reduced by 60% [74]. For covalently grafted microspheres, their zero-order release kinetic characteristics enable the steady release of chemotherapeutic drugs, resulting in a 45% reduction in the serum ALT/AST levels of tumor-bearing mice compared with the encapsulated group, which significantly alleviates the hepatotoxicity of chemotherapeutic drugs [87]. Notably, pH/enzyme dual responsive gel microspheres achieve tumor microenvironment triggered “burst release”; in the HCC interventional therapy model, this kinetic characteristic elevates the tumor necrosis rate to 80% and extends the median survival time of tumor-bearing mice from 28 days to 55 days [92]. These quantitative data clearly construct a structure-activity relationship of “gel scaffold structure-drug release kinetics HCC therapeutic outcome”, confirming that the precise regulation of sustained-release mechanisms is the core to improving therapeutic efficacy and reducing toxic side effects.

### 4.2. Synergistic Therapeutic Mediated by Gel Microspheres

**Figure 5:**
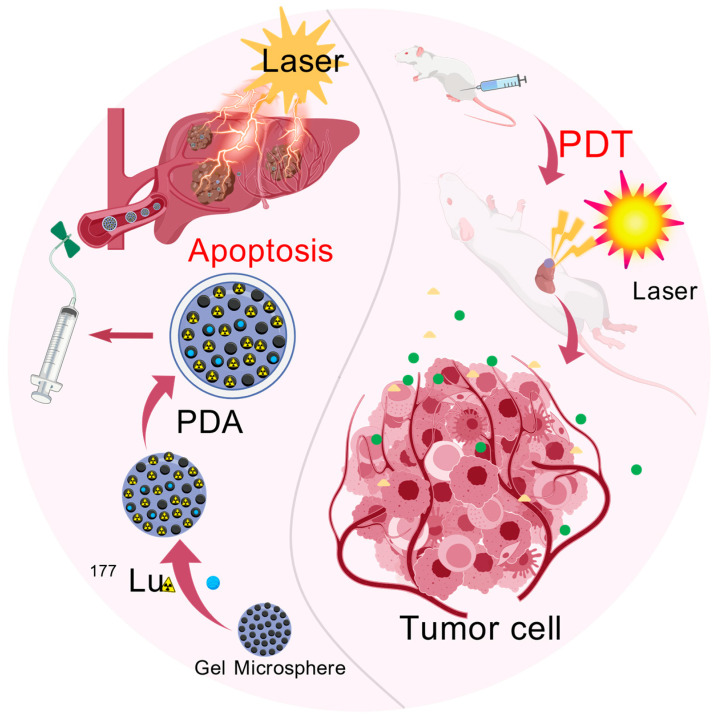
Schematic of the synergistic radiotherapy–photodynamic therapy (RT–PDT) strategy using ^177^Lu-loaded Gel microspheres for hepatocellular carcinoma treatment. Created with BioGDP.com [7].

**Figure 6:**
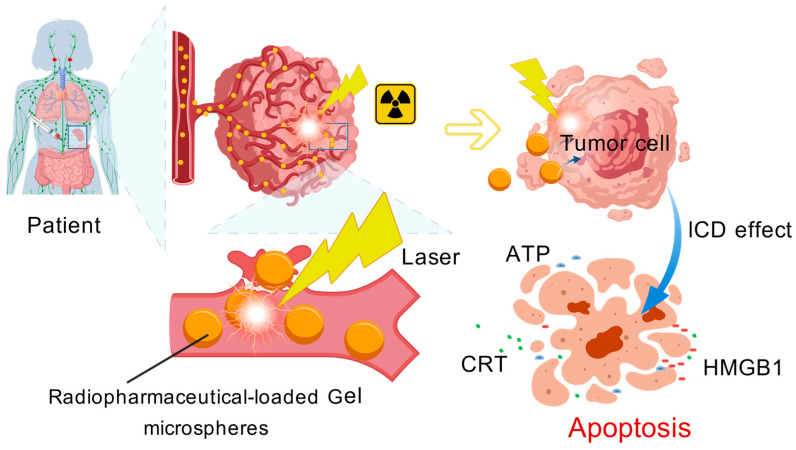
The mechanism by which transarterial radioembolization with radioactive microspheres for hepatocellular carcinoma induces immunogenic cell death in tumor cells through radiation, releasing ATP, CRT, etc., to trigger apoptosis. Created with BioGDP.com [7].

HCC has a complex pathogenesis, and single therapy is prone to drug resistance and toxic side effects [102]. Synergistic therapy has thus become a development trend in HCC treatment, with gel microspheres serving as multifunctional drug delivery scaffolds. Endowed with the core characteristics of co-encapsulating multiple therapeutic drugs, enabling targeted delivery, and achieving sustained release, gel microspheres are perfectly suited for the synergistic application of photosensitizers and other agents. They exert a synergistic enhanced effect when combined with chemotherapeutic drugs, targeted drugs, radionuclides, etc., significantly improving the therapeutic efficacy of HCC. For example, the radioactive mesoporous gel microspheres shown in Figure 5 act as the core scaffold, not only loading photosensitizers drugs but also achieving transarterial radioembolization (TARE) [103] through ^177^Lu labeling. After interventional injection, the radionuclide releases β-rays to kill tumor cells [104], and near-infrared light irradiation activates photosensitizers to generate ROS. The porous structure of the scaffold ensures the uniform loading and synchronous retention of photosensitizers and radionuclides, and the synergy between radiotherapy and PDT achieves dual killing, greatly increasing the tumor necrosis rate. Figure 6 demonstrates that radioactive gel microspheres, as scaffolds, load radionuclides and photosensitizer-related therapeutic factors: radionuclide radiotherapy induces immunogenic cell death (ICD) in tumor cells, releasing molecules such as ATP and calreticulin (CRT) to activate systemic antitumor immunity. Meanwhile, photosensitizers encapsulated in the scaffold generate ROS upon light irradiation, which can further enhance the ICD effect. The synergistic effect of RT–PDT–immunotherapy mediated by ^177^Lu-labeled gel microspheres relies on precise dose regulation, spatiotemporally matched radiation-ROS interactions and a well-defined safety margin to achieve maximum efficacy. The β-rays emitted by ^177^Lu can induce the radiolysis of water in tumor cells to generate hydroxyl radicals, increasing the yield of singlet oxygen triggered by subsequent PDT by 60%. Together, they produce a spatiotemporal synergistic effect characterized by “radiation-induced sensitization followed by PDT-mediated cytotoxic burst”, which significantly enhances the release of tumor-associated antigens and the maturation of dendritic cells (DCs) [64]. The therapeutic index of this synergistic strategy is 3.1 times that of single RT or PDT, with no obvious hepatic, renal or myelotoxicity observed [73], which fully verifies the efficacy and safety of this synergistic therapeutic strategy. In addition, gel microspheres can mediate various other synergistic modes. The synergistic effect depends not only on the reasonable combination of photosensitizers with other drugs but also on parameters including the structural design, drug encapsulation efficiency, and sustained-release rate of gel microspheres. It is necessary to optimize the preparation process in conjunction with the pathological type of HCC and the patient’s condition to maximize the value of synergistic therapy, thereby providing a safe and precise treatment regimen for HCC.

The above findings indicate that gel microspheres, by virtue of their ability to co-encapsulate multiple types of therapeutic agents, serve as an excellent multifunctional drug delivery scaffold for the synergistic therapy of HCC. They can efficiently achieve the synergistic enhancement of photosensitizers in combination with other therapies, and effectively inhibit tumor recurrence and metastasis. The full exertion of such synergistic effects highly depends not only on the rational combination of photosensitizers with other therapeutic agents but also on the core parameters of gel microspheres such as structural design, drug encapsulation efficiency and sustained-release rate. However, the translation of gel microsphere-mediated synergistic therapy for HCC from laboratory research to clinical practical application still faces numerous critical challenges inherent to gel materials themselves. Natural polymer gel microspheres suffer from poor controllability of core parameters including crosslinking degree, swelling degree and drug loading rate, as well as significant batch-to-batch variations in response performance, while synthetic polymer gel microspheres require complex surface modification to improve their biocompatibility. Moreover, the uniformity of particle size and the stability of drug encapsulation of gel microspheres are difficult to guarantee during large-scale production. In addition, the degradation rate of natural gel microspheres is prone to being out of control under the complex in vivo environment, and synthetic gel microspheres are likely to induce tissue damage due to crosslinker residues and nanoparticle toxicity. At the same time, gel microspheres have a low drug loading capacity for hydrophobic photosensitizers, and their micron-scale particle size also limits their distribution in metastatic and multifocal HCC lesions. To address the above challenges, the composite design of natural and synthetic polymer materials can be adopted to balance parameter controllability and biocompatibility, and multi-responsive gel microspheres with personalized response thresholds can be designed based on preoperative imaging and pathological test results. Preparation technologies such as microfluidics can be optimized to ensure the uniformity of particle size and the stability of drug encapsulation of gel microspheres during large-scale production. Meanwhile, molecular engineering techniques can be used to optimize the surface modification and crosslinking methods of gel microspheres, reduce crosslinker residues and nanoparticle toxicity, and improve the radionuclide labeling efficiency and radiostability of radioactive gel microspheres. Long-term animal experiments and preclinical studies should be conducted to verify their biocompatibility. Furthermore, structural modification strategies such as constructing core–shell structures and introducing hydrophilic–hydrophobic balance regulatory units can be employed to improve the drug loading capacity of gel microspheres for hydrophobic photosensitizers. Intelligent gel microspheres with adjustable particle size and real-time imaging tracking functions can be designed to adapt to the therapeutic needs of different types of HCC lesions, realize personalized clinical dosage adjustment and dynamic efficacy monitoring, thus breaking through the technical bottlenecks at the gel material level and promoting the translation of gel microsphere-mediated synergistic therapy for HCC from the laboratory to clinical application.

Although this research direction is currently in the stage of laboratory technical verification, the precise adjustability of its particle size can be initially regulated relying on mature micro-nano preparation technologies such as microfluidic chips and electrospinning. The real-time imaging tracking function can achieve basic in vivo imaging by coupling fluorescent, magnetic resonance or radionuclide-labeled probes. While clinical-grade high-resolution, non-invasive real-time tracking has not yet been realized, and there are still technical challenges such as in vivo biocompatibility and signal stability, the development of existing material molecular engineering and bioimaging technologies provides a feasible technical path for it. Such intelligent microspheres can be designed to adapt to the therapeutic needs of different types of HCC lesions, enabling personalized clinical dosage adjustment and dynamic efficacy monitoring. At the current stage, technological maturation can be gradually promoted through phased optimization of preparation processes and improvement of the in vivo stability of labeled probes, thus breaking through the technical bottlenecks at the gel material level.

### 4.3. Clinical Translation Bottlenecks and Breakthrough Pathways of Gel Microspheres in HCC Therapy

Although gel microspheres, as carriers for photodynamic therapy, exhibit advantages such as targeted delivery and sustained-release efficacy enhancement in HCC treatment, they still face significant inherent drawbacks and clinical translation challenges: natural polymer gel microspheres show large batch-to-batch performance variations, while synthetic polymer gel microspheres have insufficient biocompatibility and may exacerbate TME acidification; passive targeting fails to penetrate the deep regions of hypovascular tumors, active targeting is prone to issues like ligand shedding, and magnetic or photoresponsive types rely on external equipment, which may lead to incomplete treatment. Tumor microenvironment heterogeneity results in inconsistent drug release kinetics; ensuring particle size uniformity and drug-loading stability during large-scale production is difficult, and radioactive gel microspheres pose high challenges in radionuclide labeling and radiostability control, with their long-term biocompatibility requiring further verification. Compared to lipid nanoparticles [105] and inorganic mesoporous [106] materials, gel microspheres have lower drug-loading capacity for hydrophobic photosensitizers, and their micrometer-scale particle size limits distribution in metastatic or multifocal tumors; additionally, the lack of real-time imaging tracking functionality is unfavorable for personalized dosage adjustment.

The clinical translation of gel microspheres for HCC therapy is not only hindered by the aforementioned material and technical challenges but also constrained by multiple factors including regulatory pathways, large-scale production, GMP compliance, and economic feasibility. These combined barriers have resulted in only a handful of products achieving clinical application to date [97]. In terms of large-scale production and GMP compliance, mainstream laboratory preparation techniques such as emulsification-crosslinking and microfluidics are difficult to directly scale up. A core challenge lies in maintaining the batch-to-batch consistency of microsphere particle size and drug loading rate during mass production, while establishing a GMP-compliant sterile environment and raw material traceability system. Additionally, raw materials and manufacturing processes directly determine clinical accessibility: natural polymer microspheres, featuring low-cost raw materials and mature production processes, have a relatively low single-treatment cost, making them more suitable for popularization in primary medical institutions. In contrast, synthetic polymer microspheres and radionuclide-labeled microspheres suffer from high single-treatment costs due to complex manufacturing processes and strict quality control requirements. Furthermore, their pricing is significantly affected by nuclide supply and patent licensing, which greatly limits their clinical application in patients with advanced HCC. Therefore, to overcome these bottlenecks, future efforts should focus on three core pathways: formulating differentiated regulatory strategies and leveraging real-world data to accelerate approval processes; developing modular and continuous production technologies to improve batch-to-batch consistency and reduce costs; and adopting natural-synthetic polymer composite designs to balance performance and cost. These measures will facilitate the translation of more gel microsphere systems from laboratory research to clinical practice in HCC therapy.

## 5. Outlook and Prospects

In the future, research on gel microspheres in photodynamic therapy for hepatocellular carcinoma should focus on technical implementation and translational breakthroughs, with standardization, precision, and clinical application as the core directions: establish a standardized release testing protocol for photosensitizer-loaded gel microspheres, unify in vitro release evaluation indicators and procedures, and provide a reliable basis for preclinical screening; construct an in vitro–in vivo correlation (IVIVC) model to achieve accurate prediction of in vivo efficacy by in vitro release behavior through optimizing parameters such as gel network structure and crosslinking density; for radiolabeled gel microspheres, integrate real-time dosimetry technology with image guidance to realize dynamic monitoring and personalized adjustment of radiation dose during treatment; develop GMP-compliant large-scale preparation strategies, adopt modular continuous production technology to optimize particle size uniformity, drug-loading stability, and radionuclide labeling efficiency, while strictly controlling crosslinker residues and nanoparticle toxicity. Meanwhile, explore the synergistic delivery mode of gel microspheres with immune checkpoint inhibitors and tumor vaccines, endow carriers with more precise tumor microenvironment responsiveness and immunomodulatory functions through material molecular engineering, and promote their transformation from basic research to precise and standardized clinical application, thereby providing a more efficient and safe new option for personalized treatment of hepatocellular carcinoma.

## Figures and Tables

**Table 1:** Summary of photosensitizers for the treatment of HCC.

Photosensitizer	Advantages	Disadvantages	Gel Carrier	Ref.
Hematoporphyrin Derivative	Accumulates in tumor tissues for photodynamic therapy (PDT); enabling cancer diagnosis via fluorescence under short-wavelength light.	severe phototoxic reactions in normal tissues upon exposure.	Alginate	[12]
Protoporphyrin IX	Participates in photodynamic reactions of first-generation photosensitizers; acts as the active component of endogenous photosensitizers.	Lack of specific targeting when present alone in vivo.	Chitosan	[13]
5-Aminolevulinic Acid	Endogenous photosensitizer; selectively absorbed by proliferative cells; especially suitable for diagnosing and treating micro-lesions.	High local photosensitivity requiring strict light avoidance.	Gelatin	[14]
Meso-Tetrahydroxyphenyl Chlorin	High phototoxicity for effective tumor cell killing; good tumor selectivity to reduce normal tissue damage.	Strict control of light dose and duration.	Hyaluronic acid	[15]
Chlorophyllin a	Strong photosensitization ability; high antitumor activity; near-infrared absorption for good tissue penetration.	Relatively poor stability, requiring careful storage and handling.	Carboxymethyl cellulose	[16]
Purpurin 18	Effective tumor cell killing; good photostability for long-term activity.	Complex in vivo metabolism and difficult pharmacokinetic study.	Polyvinyl alcohol	[17]
Metallophthalocyanines	Strong absorption at 750–900 nm for deep tissue penetration; effective for melanoma; high stability.	Insufficient targeting precision, requiring modification or combination with targeting carriers.	Pluronic F127	[18]
Hypocrellin	Natural compound extracted from Shiraia bambusicola; multiple photosensitization mechanisms.	Complex extraction and purification process with limited yield.	Xanthan gum	[19]
HPPH	Effective tumor cell killing in PDT.	Interindividual response variation.	Gellan gum	[20]

**Table 2:** Summary of gel microspheres therapy for HCC.

Gel Microsphere Type	Advantages	Disadvantages	Ref.
Gelatin Sponge Microspheres (GMSs)	Excellent biocompatibility, biodegradable, no interference with subsequent treatment	Short embolization duration	[54]
Sodium Alginate Microspheres (KMG)	degraded in 3–6 months with non-toxic metabolites	Degradation rate susceptible to in vivo environment	[55]
Albumin Microspheres	Superb biodegradable, load various chemotherapeutic drugs, easy to prepare	Limited drug loading capacity	[56]
Starch Microspheres (Spherex)	Non-toxic, non-antigenic, low cost, rapidly degraded in 20–35 min, suitable for short-term embolization	Excessively fast degradation	[57]
Chitosan Microspheres	Natural biodegradable polycationic polysaccharide with mild bacteriostasis	Single surface properties	[58]
Poly(lactic-co-glycolic acid) Microspheres (PLGA)	Controllable degradation, enables long-term sustained drug release	Complex preparation process, high cost	[59]
Polylactic Acid Microspheres (PLA)	Safe degradation products, adjustable degradation time	Low drug loading efficiency	[60]
Dextran Microspheres	biodegradable, load small molecule drugs, easily metabolized by the body	unstable embolization effect possible	[61]
Yttrium-90 Resin Microspheres	Intra-radiation therapy, prolongs survival time, effective with single treatment	Extremely expensive, potential radiation risks	[62]
^177^Lu Hydrogel Microspheres	Radionuclide-labeled, suitable for combination therapy, good targeting property	High radiation safety control requirements	[63]
Magnetic Microspheres	Combine magnetic targeting and hyperthermia, enhance anti-tumor effect	Require external magnetic field equipment	[64]

**Table 3:** Comparison of relevant parameters of microspheres commonly used for HCC.

Material	Crosslinking Method	Swelling Degree	Degradation Cycle	DLR.	Ref.
GMSs	Freeze-drying crosslinking; non-toxic genipin can be used as a crosslinking agent	The lower the crosslinking degree, the higher the swelling degree	1–2 weeks	5~35%	[65]
KMG	Ion crosslinking	Ca^2+^ crosslinking: 5–20 times	3–6 months, with non-toxic metabolites	10~40%	[38]
Albumin	Photo-crosslinking can be achieved after azobenzene modification	3–10 times in the physiological environment	3–6 weeks, biodegradable	8~45%	[66]
Spherex	Sodium trimetaphosphate serves as a green crosslinking agent	20–50 times in the physiological environment	20–35 min, rapidly degraded into glucose in vivo	2~25%	[67]
Chitosan	Ion crosslinking (sodium tripolyphosphate, TPP) is the preferred method	pH-sensitive type: 10–40 times in the acidic tumor microenvironment of liver cancer	2–5 weeks, naturally biodegradable	5~40%	[68]
PLGA	Macromolecular self-assembly into spheres is achieved via the emulsification-solvent evaporation method	Hydrophobic, almost no swelling	Adjustable degradation time	1~30%	[69]
PLA	Macromolecular assembly into spheres is achieved via the emulsification-solvent evaporation method	Strongly hydrophobic, almost no swelling	Adjustable degradation time	1~25%	[70]
Dextran	Chemical crosslinking, with DVS crosslinking being milder	15–40 times in the physiological environment	2–4 weeks, no residue	3~30%	[71]
^177^Lu Hydrogel	Photo-crosslinking modification can be achieved	8–25 times in the physiological environment	4–12 weeks	5~25%	[72]
Magnetic Microspheres	Physical adsorption	Hydrophilic substrate: 5–30 times	2–12 weeks	5~45%	[73]

## Data Availability

No new data were created or analyzed in this study.

## References

[1] Liu X., Liu J., Wang X., Zou Y., Tao X., Li J., Ye M., Xu W., Deng Y., Liu L. (2025). Cancer-Secreted Exosomal miR-1246 Promotes Colorectal Cancer Liver Metastasis by Activating Hepatic Stellate Cells. Mol. Med..

[2] Reig M., Sanduzzi-Zamparelli M., Forner A., Rimola J., Ferrer-Fàbrega J., Burrel M., Garcia-Criado Á., Díaz A., Llarch N., Iserte G. (2025). BCLC Strategy for Prognosis Prediction and Treatment Recommendations: The 2026 Update. J. Hepatol..

[3] Chen M., Chen Y., Chen W., Chen X., Guo X., Yu J., Guo X., Wang M., Zhang X., Hu Q. (2025). Immune-Activated Microspheres for Enhanced Chemoembolization of Hepatocellular Carcinoma by Blocking the Adenosine A2A Receptor. Acta Biomater..

[4] Dai S., Su X., Li Z., Wang H., Liu L., Xie Y., Chai Y., Chen Y., Zhao Z., Luo B. (2025). Phosphorus-32 Microspheres: A Dual-Modality Transarterial Radioembolization Approach for Hepatocellular Carcinoma Therapy and Anti-PD1 Immunotherapy Potentiation. Mater. Today Bio.

[5] Huang Z.-M., Han X., Wang J., Gu L., Tang L., Wu S.-Y., Di T., Hou Y.-W., Lau W.Y., Jiang Y.-Q. (2024). A Prospective, Single-Arm, Phase 2 Study of Modified Transarterial Chemoembolization Using Low-Dose Chemotherapy with Blank Microspheres Plus Low-Dose Lenvatinib and Microwave Ablation in Patients with Large (≥7 cm) Unresectable Hepatocellular Carcinoma: The TALEM Trial. Liver Cancer.

[6] Porcu E.P., Salis A., Rassu G., Maestri M., Galafassi J., Bruni G., Giunchedi P., Gavini E. (2017). Engineered Polymeric Microspheres Obtained by Multi-Step Method as Potential Systems for Transarterial Embolization and Intraoperative Imaging of HCC: Preliminary Evaluation. Eur. J. Pharm. Biopharm..

[7] Jiang S., Li H., Zhang L., Mu W., Zhang Y., Chen T., Wu J., Tang H., Zheng S., Liu Y. (2025). Generic Diagramming Platform (GDP): A Comprehensive Database of High-Quality Biomedical Graphics. Nucleic Acids Res..

[8] Abdelsalam A.M., Balash A., Khedr S.M., Amin M.U., Engelhardt K.H., Preis E., Bakowsky U. (2025). Improved Photodynamic Therapy of Hepatocellular Carcinoma via Surface-Modified Protein Nanoparticles. Pharmaceutics.

[9] Ede V.G., Kate A.S. (2025). Harnessing the Potential of Pheophorbides in Photodynamic Therapy: Natural Origins, Semi-Synthetic Advances, and Future Directions. Chem. Biodivers..

[10] Zhang Y., Bo B., Qin J., Liu B., Peng H.-S. (2025). Preparation of Aggregation-Free ZnPc-Doped Nanophotosensitizers for Highly Efficient Photodynamic Therapy. Nanotechnology.

[11] Mfouo-Tynga I.S., Dias L.D., Inada N.M., Kurachi C. (2021). Features of Third Generation Photosensitizers Used in Anticancer Photodynamic Therapy: Review. Photodiagn. Photodyn. Ther..

[12] Cardoso M., Marto C.M., Paula A., Coelho A.S., Amaro I., Pineiro M., Pinho E Melo T.M.V.D., Marques Ferreira M., Botelho M.F., Carrilho E. (2024). Effectiveness of Photodynamic Therapy on Treatment Response and Survival in Patients with Recurrent Oral Squamous Cell Carcinoma: A Systematic Review. Photodiagn. Photodyn. Ther..

[13] Restrepo-Acevedo A., Murillo M.I., Orvain C., Thibaudeau C., Recberlik S., Verget L., Gómez Vidales V., Gaiddon C., Mellitzer G., Le Lagadec R. (2025). Protoporphyrin IX-Derived Ruthenium(II) Complexes for Photodynamic Therapy in Gastric Cancer Cells. Inorg. Chem..

[14] Maesaka F., Nakai Y., Yoshida T., Tomizawa M., Shimizu T., Owari T., Onishi K., Miyake M., Kuniyasu H., Fujimoto K. (2025). 5-Aminolevulinic Acid: A Novel Approach to Improving Radioresistance in Prostate Cancer. Cancers.

[15] Wang T.-H., Chou L.-F., Shen Y.-W., Lin N.-C., Shih Y.-H., Shieh T.-M. (2025). Mechanistic Insights into Temoporfin-Based Photodynamic Therapy: Ferroptosis as a Critical Regulator Under Normoxic and Hypoxic Conditions in Head and Neck Cancer. J. Photochem. Photobiol. B.

[16] Burus A., Ozcan M., Canpinar H., Bozdemir O., Zeybek N.D., Bayazit Y. (2025). The effect of the Combination Therapy with Chlorophyllin, a Glutathione Transferase P1-1 Inhibitor, and Docetaxel on Triple-Negative Breast Cancer Invasion and Metastasis In Vivo/In Vitro. Naunyn-Schmiedeberg’s Arch. Pharmacol..

[17] Yeo S., Wu H., Song Y.K., Yoon I., Lee W.K. (2025). Encapsulation of Synthesized Purpurin-18-N-Aminoimide Methyl Ester in Lipid Nanovesicles for Use as Agents in Photodynamic Cancer Therapy. J. Pharm. Sci..

[18] Alagöz T., Genç Bilgiçli H., Polat E., Pişkin H., Tüzün B., Günsel A., Erdoğmuş A., Yaraşır M.N., Bilgiçli A.T. (2025). Ag(I) Induced H-Type and pd(II) Induced J-Type Phthalocyanines to Enhance PDT Applications: Synthesis, Optical Behaviors, Photochemical/Photophysical Properties, and DFT Studies. Dalton Trans..

[19] Qu H., Sun A., Zhou Y., Ma C., Ye Y., Xu Y., Zhao H., Zhao C., Hu Y., Yang L. (2025). Hypocrellin B Exerts Its Antitumor Effect on Colorectal Cancer by Inhibiting the AKT Pathway. J. Nat. Prod..

[20] Zenga J., Awan M., Hadi Razeghi Kondelaji M., Hansen C., Shafiee S., Frei A., Foeckler J., Kuehn R., Bruening J., Massey B. (2023). Photoactivated HPPH-Liposomal Therapy for the Treatment of HPV-Negative Head and Neck Cancer. Oral Oncol..

[21] Zhang X., Deng X., Tan J., Liu H., Zhang H., Li C., Li Q., Zhou J., Xiao Z., Li J. (2024). Idarubicin-Loaded Degradable Hydrogel for TACE Therapy Enhances Anti-Tumor Immunity in Hepatocellular Carcinoma. Mater. Today Bio.

[22] Chen C., Huang X., Wang F., Yin S., Zhu Y., Han L., Chen G., Chen Z. (2023). Preparation of a Modified Silk-Based Gel/Microsphere Composite as a Potential Hepatic Arterial Embolization Agent. Biomater. Adv..

[23] Schwingel Henn G., Schmitz C., Fontana L.B., Corrêa H.V.N., Lehn D.N., Volken de Souza C.F. (2025). Water Absorption Capacity and Agricultural Utility of Biopolymer-Based Hydrogels: A Systematic Review and Meta-Analysis. ACS Polym. Au.

[24] (2025). Recent Advances in Smart Responsive Hydrogel Microspheres for Tissue Regeneration: Preparation, Characteristics and Applications. Mater. Horiz..

[25] Zhang L., Xiao Q., Xiao Z., Zhang Y., Weng H., Chen F., Xiao A. (2023). Hydrophobic Modified Agar: Structural Characterization and Application in Encapsulation and Release of Curcumin. Carbohydr. Polym..

[26] Tawfik H.O., Petreni A., Supuran C.T., El-Hamamsy M.H. (2022). Discovery of New Carbonic Anhydrase IX inhibitors as Anticancer Agents by Toning the Hydrophobic and Hydrophilic Rims of the Active Site to Encounter the Dual-Tail Approach. Eur. J. Med. Chem..

[27] Zhao W., Li Y., Tian J., Cui Q., Tang C., Yin F., Xu L., Cheng S., Fei X. (2024). Highly Stretchable Sensitive Multiscale Hydrogel Inspired by Biological Muscles for Wearing Sensors. ACS Appl. Mater. Interfaces.

[28] Liu X., Wang Y., Liang Z., Lian X., Huang D., Hu Y., Wei Y. (2023). [Progress in Preparation and Application of Sodium Alginate Microspheres]. Sheng Wu Yi Xue Gong Cheng Xue Za Zhi = J. Biomed. Eng. = Shengwu Yixue Gongchengxue Zazhi.

[29] Morshedi B., Esfandyari-Manesh M., Ghahremani M.H., Fatahi Y., Dinarvand R. (2025). Localized Co-Delivery of In-Situ Hydrogel Containing Ibrutinib-PLGA-PEG-Folate Nanoparticle and Octreotide Microsphere to Glioblastoma. Drug Deliv. Transl. Res..

[30] Egbeyemi O.I., Hatem W.A., Kober U.A., Lapitsky Y. (2024). Transforming the Stability, Encapsulation, and Sustained Release Properties of Calcium Alginate Beads Through Gel-Confined Coacervation. Langmuir.

[31] Luo H., Ma J., Yang H., Hu H., Xu Z., Jin L., Chen J., Qian C., Ju J., Zhang Y. (2025). Functionalized Photoimmune Hydrogel Microspheres for Inside-Out Eradication of Osteosarcoma via a PD-L1 PROTAC Strategy. Adv. Healthc. Mater..

[32] Gao N., Huang Y., Jing S., Zhang M., Liu E., Qiu L., Huang J., Muhitdinov B., Huang Y. (2024). Environment-Responsive Dendrobium Polysaccharide Hydrogel Embedding Manganese Microsphere as a Post-Operative Adjuvant to Boost Cascaded Immune Cycle Against Melanoma. Theranostics.

[33] Li S., Zheng W., Deng W., Li Z., Yang J., Zhang H., Dai Z., Su W., Yan Z., Xue W. (2024). Logic-Based Strategy for Spatiotemporal Release of Dual Extracellular Vesicles in Osteoarthritis Treatment. Adv. Sci..

[34] Lv Y., Wang S., Wang Y., Zhang X., Jia Q., Han S., He L. (2023). Construction and Application of Covalently Bonded CD147 Cell Membrane Chromatography Model Based on Polystyrene Microspheres. Anal. Bioanal. Chem..

[35] Zhong H., Gao X., Cheng C., Liu C., Wang Q., Han X. (2020). The Structural Characteristics of Seaweed Polysaccharides and Their Application in Gel Drug Delivery Systems. Mar. Drugs.

[36] Zafar A., Alsaidan O.A., Mujtaba M.A., Sultana S. (2025). Development of Luteolin-Loaded Calcium Alginate and Gum Tragacanth Blend Microbeads for Oral Delivery: In Vitro Characterization, Antioxidant, Antimicrobial, and Anticancer Activity Against Colon Cancer Cell Line (HT-29). Assay Drug Dev. Technol..

[37] Zhou Y., Tian J., Zhu Y., Zhang Y., Zhao X. (2024). Multilevel Chitosan-Gelatin Particles Loaded with P4HA1 siRNA Suppress Glioma Development. Drug Deliv. Transl. Res..

[38] She Z., Zhang F., Li D., Ji Y. (2025). Sodium alginate-hyaluronic acid composite particles for dox-orubicin delivery: In vitro characterization and cytotoxicity evaluation. BMC Res. Notes.

[39] Zhang Y., Liang L., Kou Q., Sun H., He Y., Li L., Shi C. (2025). Multimodal Imaging Nanoassembled Agarose Microspheres for Drug Delivery in Transarterial Chemoembolization. ACS Appl. Bio Mater..

[40] Wang L., Huang S., Kang D., Wu H., Zhu L., Mei Y., Xu C., Zhang J., Wei B., Wang H. (2025). Collagen-Decorated Drug-Loaded Polycaprolactone Microspheres for Breast Cancer Therapy In Vitro and In Vivo Model. Int. J. Biol. Macromol..

[41] Guo J., Huang J., Huang Z., Hu D., Tan H., Wang Y., Deng C., Zhu X., Zhong Z. (2025). Tumor Vessel-Adaptable Adhesive and Absorbable Microspheres for Sustainable Transarterial Chemoembolization Therapy. Nat. Commun..

[42] Cao J., Du X., Zhao H., Zhu C., Li C., Zhang X., Wei L., Ke X. (2023). Sequentially Degradable Hydrogel-Microsphere Loaded with Doxorubicin and Pioglitazone Synergistically Inhibits Cancer Stemness of Osteosarcoma. Biomed. Pharmacother..

[43] Ouyang Z., Chen X., Wang Z., Xu Y., Deng Z., Xing L., Zhang L., Hu M., Li H., Lian T. (2025). Azithromycin-Loaded PLGA Microspheres Coated with Silk Fibroin Ameliorate Inflammation and Promote Periodontal Tissue Regeneration. Regen. Biomater..

[44] Zhao Y., Chen H., Fu J., Wang A., Liu X., Jiang X. (2025). Drug-Loaded Microspheres on NIR-Responsive PLA/MXene Scaffolds: Controlled Release and Bone Tissue Regeneration. ACS Appl. Bio Mater..

[45] Deng Y., Li J., Tao R., Zhang K., Yang R., Qu Z., Zhang Y., Huang J. (2024). Molecular Engineering of Electrosprayed Hydrogel Microspheres to Achieve Synergistic Anti-Tumor Chemo-Immunotherapy with ACEA Cargo. Adv. Sci..

[46] Shi D., Ren Y., Liu Y., Yan S., Zhang Q., Hong C., Yang X., Zhao H., Zheng C., Zhao Y. (2024). Temperature-Sensitive Nanogels Combined with Polyphosphate and Cisplatin for the Enhancement of Tumor Artery Embolization by Coagulation Activation. Acta Biomater..

[47] Lai J., Azad A.K., Sulaiman W.M.A.W., Kumarasamy V., Subramaniyan V., Alshehade S.A. (2024). Alginate-Based Encapsulation Fabrication Technique for Drug Delivery: An Updated Review of Particle Type, Formulation Technique, Pharmaceutical Ingredient, and Targeted Delivery System. Pharmaceutics.

[48] Luo C., DeStefano J.J., Langlois T.J., Boyes B.E., Schuster S.A., Godinho J.M. (2021). Fundamental to Achieving Fast Separations with High Efficiency: A Review of Chromatography with Superficially Porous Particles. Biomed. Chromatogr..

[49] Yin P., Shi F., Luo M., Wu J., Zhao B., Zhang C., Shen Y., Chen Y. (2024). Preparation and Characterization of Responsive Cellulose-Based Gel Microspheres for Enhanced Oil Recovery. Gels.

[50] Yan Z., Nie Q., Liu J., Chen J., Liu Y., Lu Y., Xu M., Lin Z. (2025). Sodium Alginate/Carboxymethyl Chitosan/Gelatin-Naringenin pH-Responsive Hydrogel Beads for Oral Delivery of Traditional Chinese Herbal Medicines. Carbohydr. Polym..

[51] Liu Y., Ma W., Zhou P., Wen Q., Wen Q., Lu Y., Zhao L., Shi H., Dai J., Li J. (2023). In Situ Administration of Temperature-Sensitive Hydrogel Composite Loading Paclitaxel Microspheres and Cisplatin for the Treatment of Melanoma. Biomed. Pharmacother..

[52] Zhang X., Lian R., Fan B., Meng L., Zhang P., Zhang Y., Sun W. (2025). ROS/Enzyme Dual-Responsive Drug Delivery System for Targeted Colorectal Cancer Therapy: Synergistic Chemotherapy, Anti-Inflammatory, and Gut Microbiota Modulation. Pharmaceutics.

[53] Jin J., Chen W., Li J., Yang J., Dai R., Tang J., Li M., Chen Y., Zhang C., Liu J. (2025). Engineered Tumor Microspheres via Microfluidics and Decellularized Extracellular Matrix for High-Throughput Organoid-Based Drug Screening. Biofabrication.

[54] Yuan G.-S., Zhang L.-L., Chen Z.-T., Zhang C.-J., Tian S.-H., Gong M.-X., Wang P., Guo L., Shao N., Liu B. (2023). Comparison of Ethanol-Soaked Gelatin Sponge and Microspheres for Hepatic Arterioportal Fistulas Embolization in Hepatic Cellular Carcinoma. World J. Gastrointest. Oncol..

[55] Ciarleglio G., Russo T., Toto E., Santonicola M.G. (2024). Fabrication of Alginate/Ozoile Gel Microspheres by Electrospray Process. Gels.

[56] Li F., Cai R., Ye Z., Yang L., Qiu X., Sun X. (2025). Human Serum Albumin Microspheres Synchronously Loaded with ZIF-8 and Triptolide (TP) for the Treatment of Intrahepatic Cholangiocarcinoma. J. Biomater. Appl..

[57] Vogl T.J., Lilienthal C., Gruber-Rouh T., Afraz Z., Adwan H. (2023). Degradable Starch Microspheres Transarterial Chemoembolization with or Without Lipiodol for Liver Metastases from Pancreatic Cancer: A Prospective Randomized Trial. Cancers.

[58] Lv Y., Su L., Zhao Z., Zhao J., Su H., Zhang Z., Wang Y. (2023). Chitosan Microspheres Loaded with Curcumin and Gallic Acid: Modified Synthesis, Sustainable Slow Release, and Enhanced Biological Property. Curr. Microbiol..

[59] Chen W., Li H., Zhang X., Sang Y., Nie Z. (2024). Microfluidic Preparation of Monodisperse PLGA-PEG/PLGA Microspheres with Controllable Morphology for Drug Release. Lab Chip.

[60] Thornell T.L., Wedgeworth D.N., Antwine M.D., Burroughs J.F. (2024). Influence of Carbonyl Iron Particles (CIP) and Glass Microspheres on Thermal Properties of Poly(Lactic Acid) (PLA). Polymers.

[61] Thodikayil A.T., Hemlata H., Murali N., Minocha S., Betal S., Saha S. (2026). Nano Enabled Dual-Responsive Drug Carrier Originated from Acetalated Dextran/Carboxylated Nanocellulose-Based Core-Shell Microspheres. ACS Biomater. Sci. Eng..

[62] Agirrezabal I., Bouattour M., Pinato D.J., D’ALessio A., Brennan V.K., Carion P.L., Shergill S., Amoury N., Vilgrain V. (2023). Efficacy of Transarterial Radioembolization Using Y-90 Resin Microspheres Versus Atezolizumab-Bevacizumab in Unresectable Hepatocellular Carcinoma: A Matching-Adjusted Indirect Comparison. Eur. J. Cancer.

[63] Sun J., Sun X., Yin L., Jin S., Huang Q., Dong Y., Gu X., Zhang Y., Jin Y., Zhu R. (2025). Dual Functional Radioactive Gel-Microspheres for Combinatorial Radioembolization and Photothermal Therapy of Hepatocellular Carcinoma. Adv. Healthc. Mater..

[64] Wu M., Wang D., Qin Y., Qi X., Huang Q., Sun X., Jin Y., Zhu R., Wang G., Rong P. (2025). Radio-Magnetic Dual-Functional Microspheres for Magnetic Hyperthermia Therapy Combined with Radioembolization of Hepatocellular Carcinoma. Mater. Today Bio.

[65] Zhao X., Wang Z., Zhao G., Zhang Y., Ji M. (2020). Experimental Study on Embolization of Rabbit Renal Artery with Gelatin Sponge Microspheres. J. Cancer Res. Ther..

[66] Brunson C.P., McGregor H.J., Hennemeyer C.T., Patel M.V., Woodhead G.J., Young S.J. (2024). Measurement of the Tumor-to-Normal Ratio for Radioembolization of Hepatocellular Carcinoma: A Prospective Study Comparing 2-Dimensional Perfusion Angiography, Technetium-99m Macroaggregated Albumin, and Yttrium-90 SPECT/CT. J. Vasc. Interv. Radiol..

[67] Collettini F., Andrašina T., Reimer P., Schima W., Stroszczynski C., Lamprecht Y., Auer T.A., Rohan T., Wildgruber M., Gebauer B. (2025). Degradable Starch Microspheres Transarterial Chemoembolization (DSM-TACE) in Patients with Unresectable Hepatocellular Carcinoma: Results from the Prospective Multicenter Observational HepaStar Trial. Eur. Radiol..

[68] Zhang H., Pan X., Wu Q., Wu Y., Zheng N., Ning S., Zeng D., Chen L., Li W., Wang J. (2025). Synthesis and Characterization of Functional Chitosan-Based Microspheres as Biodegradable Yttrium-90 Delivery System for Radioembolization Therapy. Int. J. Biol. Macromol..

[69] Li P., Zhai Z., Fang J., Wang R., Li W., Wang B., Wang J., Zhu J., Bing F., Pan Q. (2024). PLGA Micro/Nanoparticle Vaccination Elicits Non-Tumor Antigen Specific Resident Memory CD8+ T Cell Protection from Hepatocellular Carcinoma. Nanoscale.

[70] Kim H.-C., Choi J.W. (2024). Comparative Study Between Embosphere^®^ and Marine gel^®^ as Embolic Agents for Chemoembolization of Hepatocellular Carcinoma. World J. Gastrointest. Oncol..

[71] Kim D.-H., Choy T., Huang S., Green R.M., Omary R.A., Larson A.C. (2014). Microfluidic Fabrication of 6-Methoxyethylamino Numonafide-Eluting Magnetic Microspheres. Acta Biomater..

[72] Li X., Zhong B., Jiang N., Huang J., Hu D., Zhou R., Zeng J., Shu W., Duan G., Wu S. (2025). Disphosphate Based Hydrogel Microspheres for Targeted Transarterial Radioembolization and Chemoembolization Therapies. J. Adv. Res..

[73] Yu Z., He Y., Wang M., Shen J., Wang D., Yu A., Gu J., Hong Z., Pei Z., Sun X. (2025). Enhanced Magnetic Thermal Ablation Combined with Immunotherapy for Hepatocellular Carcinoma Using Engineering Microspheres. Mater. Today Bio.

[74] Zhu S., Shou X., Kuang G., Kong X., Sun W., Zhang Q., Xia J. (2025). Stimuli-Responsive Hydrogel Microspheres Encapsulated with Tumor-Cell-Derived Microparticles for Malignant Ascites Treatment. Acta Biomater..

[75] Wang Y., Zeng Y., Liu G. (2025). Advanced Microsphere and Hydrogel Platforms for Precision Interventional Therapy of Hepatocellular Carcinoma. Chemistry.

[76] Zhang Q., Wan T., Jin G., Xu S. (2024). pH-Responsive Chitosan-Mediated Spherical Mesoporous Silica Microspheres for High Loading and Controlled Delivery of 5-Fluorouracil. Carbohydr. Res..

[77] Li K., Zhao H., He X., Sun C., Xu R., Li Q. (2024). Ca^2+^-Mediated Chitosan/Sodium Alginate Encapsulated Red Monascus Pigment Hydrogel Beads: Preparation, Characterization and Release Kinetics. Int. J. Biol. Macromol..

[78] Ye H., Zhang R., Zhang C., Xia Y., Jin L. (2024). Advances in Hyaluronic Acid: Bioactivity, Complexed Biomaterials and Biological Application: A Review. Asian J. Surg..

[79] Higashi S.L., Isogami A., Takahashi J., Shibata A., Hirosawa K.M., Suzuki K.G.N., Sawada S., Tsukiji S., Matsuura K., Ikeda M. (2022). Construction of a Reduction-Responsive DNA Microsphere Using a Reduction-Cleavable Spacer Based on a Nitrobenzene Scaffold. Chem.-Asian J..

[80] Ambrosio J.A.R., Marmo V.L.M., Gonçalves E.P., Pinto J.G., Ferreira-Strixino J., Raniero L.J., Beltrame M., Simioni A.R. (2023). Hydroxyapatite Microspheres Used as a Drug Delivery System for Gliosarcoma Strain 9l/lacz Treatment by Photodynamic Therapy Protocols. Photodiagn. Photodyn. Ther..

[81] Zhang H., Li S., You J., Yin L., Wang D., Zheng W., Yuan Q., Jin Y., Sun X. (2026). Preparation of High-Performance PVA@Fe_3_O_4_ Magnetothermal Microspheres for Precise Ablation of Orthotopic Hepatocellular Carcinoma in a Rabbit Model. Int. J. Pharm..

[82] Lin X., Deng S., Fu T., Lei Y., Wang Y., Yao J., Lu Y., Huang Y., Shang J., Chen J. (2025). Hyaluronic Acid-Based Hydrogel Microspheres with Multi-Responsive Properties for Antibacterial Therapy and Bone Regeneration in Staphylococcus Aureus-Infected Skull Defects. Mater. Today Bio.

[83] Li X., He G., Su F., Chu Z., Xu L., Zhang Y., Zhou J., Ding Y. (2020). Regorafenib-Loaded Poly (Lactide-Co-Glycolide) Microspheres Designed to Improve Transarterial Chemoembolization Therapy for Hepatocellular Carcinoma. Asian J. Pharm. Sci..

[84] Sulaiman S., Rani R.A., Mohamad Yahaya N.H., Tabata Y., Hiraoka Y., Seet W.T., Ng M.H. (2022). Physical and Natural Crosslinking Approaches on Three-Dimensional Gelatin Microspheres for Cartilage Regeneration. Tissue Eng. Part C Methods.

[85] Faille C., Lemy C., Allion-Maurer A., Zoueshtiagh F. (2019). Evaluation of the Hydrophobic Properties of Latex Microspheres and Bacillus Spores. Influence of the Particle Size on the Data Obtained by the MATH Method (Microbial Adhesion to Hydrocarbons). Colloids Surf. B Biointerfaces.

[86] Xiao L., Li Y., Geng R., Chen L., Yang P., Li M., Luo X., Yang Y., Li L., Cai H. (2023). Polymer Composite Microspheres Loading 177Lu Radionuclide for Interventional Radioembolization Therapy and Real-Time SPECT Imaging of Hepatic Cancer. Biomater. Res..

[87] Yun X., Chen W., Zhang J., Dong T. (2023). Colorimetric Porous Microspheres of Natural Sodium Alginate for Chilled Pork Visual Monitoring. Int. J. Biol. Macromol..

[88] Li L., Du Y., Yin Z., Li L., Peng H., Zheng H., Yang A., Li H., Lv G. (2020). Preparation and the Hemostatic Property Study of Porous Gelatin Microspheres Both In Vitro and In Vivo. Colloids Surf. B Biointerfaces.

[89] Shi S., Zhang Y., Huang J., Wang Z., Lv W., Li X., Wang Y., Huang C., Liu H. (2024). Drug Delivery Particles for Targeted Imaging-Guided Photothermal/Chemotherapy Synergy Cancer Therapy. Heliyon.

[90] García-Briega M.I., Plá-Salom J., Clara-Trujillo S., Tolosa L., Cordón L., Sempere A., Ribelles J.L.G. (2025). Co-Culture of Multiple Myeloma Cell Lines and Bone Marrow Mesenchymal Stem Cells in a 3D Microgel Environment. Biomater. Adv..

[91] Li Z., Ye B.C., Xie R.Y., Wang Y.Y., Zhang H.T., Hu X., Li Y., Wu P.L., Ge P., Yu B.L. (2022). [Analysis of Curative Effects of Chemoembolization with Drug-Loaded Microspheres of Different Particle Sizes for the Treatment of Hepatocellular Carcinoma]. Zhonghua Gan Zang Bing Za Zhi = Zhonghua Ganzangbing Zazhi = Chin. J. Hepatol..

[92] Gupta A., Kulkarni S., Soman S., Saha M., Kulkarni J., Rana K., Dhas N., Ayesha Farhana S., Kumar Tiyyagura P., Pandey A. (2024). Breaking Barriers in Cancer Management: The Promising Role of Microsphere Conjugates in Cancer Diagnosis and Therapy. Int. J. Pharm..

[93] Zhang M., Hu W., Cai C., Wu Y., Li J., Dong S. (2022). Advanced Application of Stimuli-Responsive Drug Delivery System for Inflammatory Arthritis Treatment. Mater. Today Bio.

[94] Chen X., Luo F., Yuan M., Bai C., Chen Q., Zhang K., Fan Y., Cao C., Wang L., Ye F. (2024). Alginate/Chitosan-Based Hemostatic Microspheres Loaded with Doxorubicin Liposomes for Postoperative Local Drug Delivery in Solid Tumor. Int. J. Biol. Macromol..

[95] Xiong B., Shao X., Fang G., Dong M., Han H., Li Q. (2025). Porous PLGA Microspheres for the Inhalation Delivery of Icariin and miR-23b in the Treatment of Metastatic Lung Cancer. Asian J. Pharm. Sci..

[96] Ayyanaar S., Kesavan M.P. (2023). Magnetic Iron Oxide Nanoparticles@lecithin/Poly (L-Lactic Acid) Microspheres for Targeted Drug Release in Cancer Therapy. Int. J. Biol. Macromol..

[97] Ciampi-Dopazo J.J., Ruiz Villaverde G., Espejo J.J., García Marcos R., Pérez Enguix D., Pisoni S., Martínez-Rodrigo J.J., Navarro Vergara P., Pardo Moreno P., Rodríguez-Fernández A. (2025). Health Outcomes and Resource Consumption Analysis of Radioembolization with Y90 Glass Microspheres (TARE-Y90) Versus Transarterial Chemoembolization with Irinotecan (DEBIRI) in Patients with Liver Metastases from Colorectal Cancer in Spain. Diagnostics.

[98] Wang Y., Ren Z., Wu H., Cao Y., Yu B., Cong H., Shen Y. (2024). Immobilized Drugs on Dual-Mode Imaging Ag2S/BaSO4/PVA Embolic Microspheres for Precise Localization, Rapid Embolization, and Local Antitumor Therapy. ACS Appl. Mater. Interfaces.

[99] Jaroch D.B., Liu Y., Kim A.Y., Katz S.C., Cox B.F., Hullinger T.G. (2024). Intra-Arterial Pressure-Enabled Drug Delivery Significantly Increases Penetration of Glass Microspheres in a Porcine Liver Tumor Model. J. Vasc. Interv. Radiol..

[100] Han H., Wang S., Shahbazi M.-A., Du Y., Zuhorn I.S., Li J., Chen J., Chen Y., Bártolo R., Cui W. (2025). Local Glycolysis-Modulating Hydrogel Microspheres for a Combined Anti-Tumor and Anti-Metastasis Strategy Through Metabolic Trapping Strategy. J. Control. Release.

[101] Zheng Z., Ma M., Han X., Li X., Huang J., Zhao Y., Liu H., Kang J., Kong X., Sun G. (2023). Idarubicin-Loaded Biodegradable Microspheres Enhance Sensitivity to Anti-PD1 Immunotherapy in Transcatheter Arterial Chemoembolization of Hepatocellular Carcinoma. Acta Biomater..

[102] Choi S.J., Lee S., Choi H., Ko M.J., Kim D., Kim D.-H. (2024). Development of Injectable Colloidal Solution Forming an In Situ Hydrogel for Tumor Ablation. Biomater. Sci..

[103] Dong Y., Yin L., Huang J., Hu D., Sun J., Zhang Z., Li Z., Zhong B.-Y., Zhu R., Wang G. (2024). 99mTc/90Y Radiolabeled Biodegradable Gel Microspheres for Lung Shutting Fraction Assessment and Radioembolization in Hepatocellular Carcinoma Theranostics. Mater. Today Bio.

[104] Shukla J., Chopra S., Kaur K., Chakraborty S., Singh H., Duseja A., Kalra N., Mittal B.R. (2024). 177 Lu-Microspheres Selective Intra-Arterial Radionuclide Therapy: A Facile and Biocompatible Permanent Micro-Seed Implants for Unresectable Hepatocellular Carcinoma. Clin. Nucl. Med..

[105] Wang N., Lu S., Cao Z., Li H., Xu J., Zhou Q., Yin H., Qian Q., Zhang X., Tao M. (2025). Pyruvate Metabolism Enzyme DLAT Promotes Tumorigenesis by Suppressing Leucine Catabolism. Cell Metab..

[106] Li X., Liu Y., Ke J., Wang Z., Han M., Wang N., Miao Q., Shao B., Zhou D., Yan F. (2024). Enhancing Radiofrequency Ablation for Hepatocellular Carcinoma: Nano-Epidrug Effects on Immune Modulation and Antigenicity Restoration. Adv. Mater..

